# Do Social Exchange Relationships Influence Total-Quality-Management Involvement? Evidence from Frontline Employees of International Hotels

**DOI:** 10.3390/bs13121013

**Published:** 2023-12-14

**Authors:** Chung-Jen Wang

**Affiliations:** Department of Hotel and Restaurant Management, National Pingtung University of Science and Technology, Pingtung 912301, Taiwan; wchungzen@gmail.com; Tel.: +886-8770-3202 (ext. 7545)

**Keywords:** LMX, TMX, TQM involvement, self-efficacy, job satisfaction, hospitality, human health, sustainable growth

## Abstract

This study delves into the assessment of the quality of social exchange relationships in the workplace, specifically focusing on leader–member exchange (LMX) and team–member exchange (TMX), to forecast employee Total Quality Management (TQM) involvement within the hospitality industry. Employing Structural Equation Modeling (SEM), the study evaluates multiple hypotheses, utilizing data collected from 811 frontline employees in international tourist hotels located in Taiwan. The findings demonstrate that both LMX and TMX exhibit direct positive influences on employee TQM involvement. Moreover, through mediated path analyses, it is evident that both LMX and TMX exert indirect positive impacts on employee TQM involvement, by means of self-efficacy and job satisfaction. These results elucidate a clear causal chain mechanism underlying the behavior of employee TQM involvement within such social relationships. The study’s revelations carry significant implications for the hospitality industry, shedding light on the nuanced dynamics of these social relationships and their impact on employee behavior concerning TQM involvement. The discussions encompassing the implications of these findings in the realm of hospitality are thoroughly explored and detailed.

## 1. Introduction

Although total quality management (TQM) has been one the most important strategic resources that can help firms achieve sustainable competitive advantage in a dynamic global economy [1], few prior studies have paid attention to factors that influence employee TQM involvement. Employee involvement means all employees in an organization being involved in having information, making decisions and having the effective power to do so [2,3]. Since TQM needs to involve employees in each quality process, organizations with successful implementation of employee involvement can thus build an environment that fosters mutual communication, shares corporate values, integrate different opinions and leads to business excellence. As a result, that participation of employees is the key component for the success of TQM [4].

A substantial body of evidence now points towards the quality of social exchange relationships within organizations as a significant predictor of both employee performance and job satisfaction [5,6,7]. According to Blau [8], social exchange theory refers to enduring, unspecified obligations that arise when one individual provides assistance to another, with the expectation of reciprocation [9]. Liao, Liu, and Loi [10] suggest that the interpersonal relationships between each individual and their leaders and coworkers in organizations compose a social system. Therefore, the reciprocal exchanges with a vertical dyad linkage between the employee and his or her leader are conceptualized as leader–member exchanges (LMX) [11], and the reciprocal exchanges with a horizontal dyad linkage between the employee and his or her coworkers are defined as team–member exchanges (TMX) [12]. From the perspective of human needs and motivation, these interpersonal relationships in a managerial and organizational context are composed of ethical core values such as trust, which leads to employee commitment, satisfaction and loyalty [13]. While many prior studies have examined how LMX or TMX may influence organizational commitment, task performance, and job satisfaction [5,14,15,16], few scholars have examined the impacts of these together. More specifically, if an organization is looking for the employee TQM involvement, these social exchange relationships such as LMX and TMX are both proposed to be important antecedents that influence employee self-efficacy and satisfaction, which are related to their work quality and job performance [17].

According to social cognitive theory, self-efficacy is an individual’s belief that he or she is able to perform an assigned task well [18], and of a high quality, with a leader and team mates who can all help to enhance employee self-efficacy, and thus promote performance [6,7,10]. Therefore, successful implementation of TQM needs employee involvement with high self-efficacy to find problems relating to quality, and generate useful and novel ideas, with the creativity for problem solving. In addition, in Deming’s TQM method [19], continual development of individual abilities can foster quality performance, and self-efficacy is regarded as one’s belief in this capability, which continuously promotes employees’ activities for quality improvement [20]. Since the nature of TQM activities is to seek continuous quality improvement, the participation of these high-self-efficacy employees can assist in solving problems relating quality, and create business profits for organizations. In addition, if employees have good relationships with leaders and coworkers, they will have more satisfaction regarding their jobs. Most important of all, these satisfied employees tend to have more willingness to stay in the organization, to participate in the TQM activities, to find solutions for quality improvement, and to contribute their efforts to work with their leaders and members in meeting the organizational quality goals.

Over the last decade, companies in the hospitality sector have been facing more pressure to maintain competitive advantages, and the implementation of TQM is critical for survival in a business environment [21,22,23,24]. Moreover, since the nature of hospitality is labor intensive, and frontline employees have the most direct contact with customers, their involvement in TQM is regarded as a consequence of social interaction with leaders and members, and can thus help the company to increase customer satisfaction and decrease advertising costs, and lead to a better business performance [25,26,27,28,29]. The distinctive traits of the hospitality industry underscore the crucial role of TQM, given its intricate service delivery, extensive customer engagement, and heavy dependence on human resources. Accordingly, this paper delves into the often-neglected factors that influence TQM involvement among employees, a pivotal aspect which is crucial for organizational success and competitiveness in today’s dynamic global economy. The subsequent sections provide an in-depth theoretical exploration of the impact of social exchange relationships, particularly LMX and TMX, on employee TQM involvement within the hospitality industry. Furthermore, these sections meticulously scrutinize the significant mediating roles of self-efficacy and job satisfaction. These mediating factors offer valuable insights into the underlying mechanisms through which LMX and TMX exert their influence on TQM participation among frontline employees. Later sections of this paper outline the methodological approach, including the use of Structural Equation Modeling (SEM) to analyze data collected from 811 frontline employees in international tourist hotels in Taiwan. It details the survey instruments, data collection procedures, and the analytical techniques employed to test the hypotheses. By developing and examining theories on the relationship between LMX, TMX, self-efficacy, job satisfaction and TQM involvement, this research offers a new perspective to the literature on the vertical- and horizontal-dyad linkages of TQM contexts.

## 2. Conceptual Development and Hypotheses

**Figure 1:**
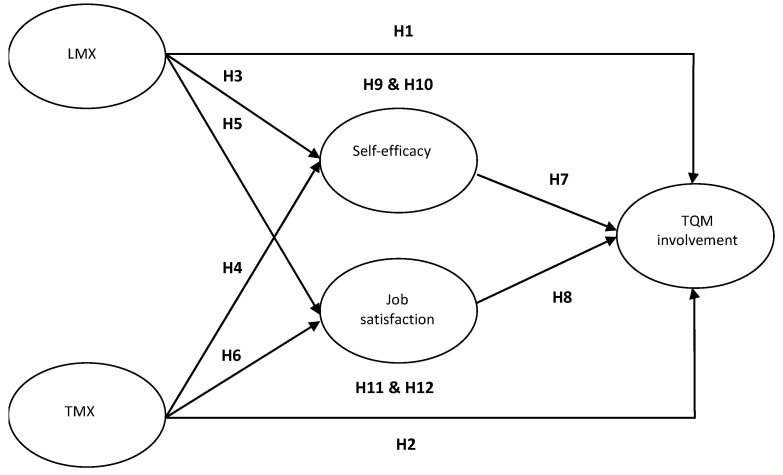
The theoretical model.

In Figure 1, this work first investigates the quality of exchange relationships with leaders and coworkers, as predictors of employee TQM involvement. It then considers the relationships among LMX, TMX, self-efficacy, job satisfaction and TQM involvement. Finally, it integrates work on these reciprocal exchange relationships, arguing that self-efficacy and job satisfaction both mediate the effects of LMX and TMX on TQM involvement.

### 2.1. The Relationships among LMX, TMX and TQM Involvement

Graen [11] proposed that the vertical-dyad linkage between the employee and his or her leader are conceptualized as LMX. From the perspective of LMX, leaders develop different exchange relationships with each employee, and high-quality LMX is characterized by vertical-dyad linkages of mutual trust, loyalty, and reciprocal influence [30,31]. Since the concept of total quality needs employees in an organization who have a common goal for quality improvement [4], the linkage between leader and followers is critical for implementation of TQM. That is, this reciprocal trust is the core value of TQM, which creates a friendly environment for better relationship marketing, as well as organizational learning in quality management [32]. Moreover, this positive relationship also helps to create an atmosphere that encourages all employees in an organization to become involved in TQM-related activities. Especially in the field of hospitality, employees can thus deliver quality services to customers, and maintain their loyalty when they feel they are supported by their leaders [33,34,35,36,37,38]. From an external marketing perspective, this customer loyalty can be linked to business profitability from these high-value services. For example, Warwood and Roberts [39] investigated the development of leadership and subordinate TQM involvement based on two surveys in the UK, and the results showed support for a positive relationship between these two variables. Similarly, Aksu [40] revealed that high-quality relationships with leader can enhance employee TQM involvement, based on the data of 319 administrators in Turkey. On the other hand, TMX represents the overall quality of the relationships between an individual and his or her coworkers [12]. As with LMX, low-quality TMX will limit the exchanges of resources, while high-quality TMX increases them via sharing, cooperation, and social rewards [30]. Therefore, if an employee has good relationships with his or her partners, team members will be more willing to provide assistance and feedback about each other’s work, thus enhancing individual performance by providing evidence of mastery of experiences [41]. In the hospitality sector, this positive atmosphere with coworkers is the key to enhancing employee involvement in TQM, for adopting more innovative problem-solving methods and undertaking more challenging activities, and thus meeting the expectation of customers [2,42,43]. Since employees are the most valuable asset in organizations, the quality of their motivations in having information and making decisions can ultimately contribute to creating quality improvement [4]. For instance, Chang and Sinclair [44] carried out a case study analysis, and found that high-quality relationships with team members can have positive relationship with employee TQM-related performance. In addition, Sila and Ebrahimpour [45] reported that higher levels of teamwork quality contribute to greater employee involvement in TQM, based on an across-countries survey. Accordingly, both high-LMX and high-TMX employees are expected to have high-TQM involvement behavior, and we propose the first two hypotheses:

**Hypothesis** **1.** *LMX has a positive direct effect on employee TQM involvement.*

**Hypothesis** **2.** *TMX has a positive direct effect on employee TQM involvement.*

### 2.2. The Relationships between LMX and TMX, and Self-Efficacy

Self-efficacy, a central tenet of social cognitive theory [18,41], is critical in driving employees’ confidence and beliefs in their capabilities, significantly affecting their performance and service quality. High self-efficacy, nurtured by strong relationships with leaders and coworkers, encourages employee involvement in TQM activities [40,46]. Studies have consistently shown the positive impact of high-quality LMX and TMX on employee self-efficacy, leading to increased TQM involvement [46,47]. Therefore, the relationship between LMX and self-efficacy, as well as TMX and self-efficacy, forms the foundation for successful TQM practices in an organization. LMX represents the association between leaders and employees, while TMX signifies the rapport between coworkers. High-quality relationships with both leaders and coworkers significantly contribute to enhancing employee self-efficacy. These strong relationships positively influence employees’ involvement in TQM activities, and result in the generation of innovative solutions and improved work quality [44]. Moreover, this enhanced self-efficacy fosters employee satisfaction, thereby increasing their willingness to contribute to TQM practices and achieve organizational quality goals [40]. In the hospitality sector, the interaction between LMX, TMX, and self-efficacy becomes a critical factor, influencing customer satisfaction and organizational performance. Enhanced social interactions and fulfillment of employee needs foster self-efficacy, encouraging employees to contribute more to organizational goals, meet quality standards, and contribute to customer satisfaction. We thus propose the following hypotheses:

**Hypothesis** **3.** *LMX has a positive direct effect on self-efficacy.*

**Hypothesis** **4.** *TMX has a positive direct effect on self-efficacy.*

### 2.3. The Relationships between LMX and TMX, and Job Satisfaction

The association between LMX and job satisfaction, as well as TMX and job satisfaction, reveals critical factors in TQM involvement among employees [46], since LMX depicts the relationship between leaders and individual employees, emphasizing trust, loyalty, and reciprocal influence. High-quality LMX involves favorable treatment from leaders, providing resources, challenging tasks, training, and promotional opportunities, which enhance employee aspirations, job satisfaction, and commitment [33]. In addition, TMX represents the overall quality of relationships among coworkers, facilitating support and feedback in the workplace. Strong TMX relationships are linked to increased job satisfaction and overall satisfaction among team members, and foster a positive atmosphere that encourages innovative problem-solving, more challenging activities, and meeting customer expectations, vital aspects in the hospitality sector [10,48]. Both high-quality LMX and TMX relationships contribute to employee involvement in TQM activities, through increased job satisfaction [7]. The fulfillment of employee needs—such as recognition, belonging, and a sense of identity from both leaders and team members—enhances job satisfaction and overall quality of working life. Satisfied employees are more willing to participate in TQM activities, seek solutions for quality improvement, and contribute their efforts toward meeting organizational quality goals. Studies consistently highlight the positive relationship between high-quality LMX and TMX and job satisfaction [29,30,49], and we propose the following hypotheses:

**Hypothesis** **5.** *LMX has a positive direct effect on job satisfaction.*

**Hypothesis** **6.** *TMX has a positive direct effect on job satisfaction.*

### 2.4. The Relationships between Self-Efficacy and Job Satisfaction, and TQM Involvement

Continuous development of individual capabilities, a fundamental principle in Deming’s TQM method, is associated with higher self-efficacy, leading to an increase in the quality of performance [45]. Employees with high self-efficacy tend to be more satisfied with their work, and contribute more to the organization. In the hospitality sector, successful TQM implementation requires employees with high self-efficacy, generating creative solutions and novel ideas for problem-solving [50]. Furthermore, in a supportive work atmosphere where employees feel supported and cared for by their leaders and coworkers, self-efficacy regarding the quality of performance is enhanced. Studies consistently support the positive relationship between high self-efficacy and employee involvement in TQM [21,51]. Additionally, job satisfaction plays a vital role in influencing TQM involvement. Job satisfaction is associated with the fulfillment of various needs, such as a sense of belonging, identity, and recognition from both leaders and team members [44,45]. Satisfied employees are more motivated to contribute to the organization’s quality goals. In the hospitality industry, job satisfaction is particularly relevant, as it can lead to customer satisfaction, reduced advertising costs, and improved business performance [46]. As a result, the relationship between self-efficacy and TQM involvement, as well as job satisfaction and TQM involvement, is substantial, and critical in influencing employees’ engagement in TQM activities within organizations. We thus propose the following hypotheses:

**Hypothesis** **7.** *Self-efficacy has a positive direct effect on TQM involvement.*

**Hypothesis** **8.** *Job satisfaction has a positive direct effect on TQM involvement.*

### 2.5. The Mediating Roles of Self-Efficacy and Job Satisfaction

Self-efficacy is a central component of social cognitive theory, and it affects employee goals, efforts and task persistence [38,50]. Bandura [52] posited that four types of experience determine the development of individual self-efficacy: mastery experience (enactive attainment), vicarious experience (modeling), social persuasions and physiological factors, and he defined self-efficacy as an individual’s belief in his or her abilities to mobilize the motivation and cognitive resources in order to take action to carry out specific tasks. As for TQM relative activities, high-self-efficacy employees possess greater confidence and stronger beliefs that they are able to succeed, compared to employees who have a fear of failure, and thus have significantly positive effects on service quality [6,20]. Moreover, in Deming’s TQM method [19], continual development of individual capabilities can enrich quality performance, and self-efficacy is regarded as one’s belief in this capability, which continuously promote employees’ participation in activities regarding quality improvement. Consequently, such individuals will be happier with their work, because they have more confidence in their capabilities, and so will contribute more to the organization [53]. In the field of hospitality, successful TQM also needs the involvement of employees with high self-efficacy, to generate useful and novel ideas, with a creativity for problem solving [2]. From an internal marketing perspective, when employees have good relationships with their leaders and team members, their self-efficacy for involvement in TQM-related activities will be fostered. Moreover, as an employee’s involvement in TQM involves social interaction with people, it is only when individuals feel they are supported and cared for by leaders and coworkers that their abilities regarding the quality of their performance can be increased because they enjoy this work atmosphere and have more willingness to work with these members. As a result, this mutual trust between employee and leaders, as well as employee and teammates, can help to promote employees’ self-efficacy, and thus enhance their TQM involvement [32]. A number of studies have provided evidence of this; for example, Kamdar and Van Dyne [54] examined how LMX and employee personality at work can predict task performance, and they found that high-quality social exchange relationships can improve the quality of performance, from matched data of 230 employees. Liden et al. [6] carried out a field investigation of 337 employees and their team members, and the result reveals that TMX is positively related to service performance. Liao et al. [10] found that both LMX and TMX have positive effects on employee self-efficacy, based on multisource data from 828 employees in 116 teams. In addition, Tang et al. [2] also revealed that self-efficacy is positively related to employee TQM involvement, from a survey over 10 years. Since LMX represents the quality of the relationships with leaders, while TMX demonstrates the quality of the relationships among coworkers, we argue that both high-quality LMX and high-quality TMX are expected to improve employee TQM involvement, via high self-efficacy. Therefore, we present the following hypotheses:

**Hypothesis** **9.** *LMX has a positive indirect effect on employee TQM involvement, via self-efficacy.*

**Hypothesis** **10.** *TMX has a positive indirect effect on employee TQM involvement, via self-efficacy.*

In addition, job satisfaction is an important phenomenon of organizational behavior, and is associated with employee turnover intention and work performance [49]. The theory of exchange proposes that satisfaction of interests within social interaction depends on how they are determined in the social experiences [8,55]. With high-quality LMX relationships, leaders provide substantial resources, more challenging tasks, and training and promotion opportunities to specific employees [30,56]. These favorable treatments increase employee aspirations to take on more responsibility, and lead to greater job satisfaction and employee commitment. On the other hand, members in a work team define their roles by reciprocal, reinforcing interactions with coworkers, based on the capabilities, interests, and needs of the team and its members [57]. By providing appropriate work-related social support and feedback, coworkers in a team provide the necessary conditions for the enhanced perceptions of competence that are needed to complete the assigned tasks [6]. TMX quality has thus been linked to employee job satisfaction, while higher-quality relationships with team members are more likely to lead to greater overall satisfaction [12]. Most important of all, employee involvement in TQM activities and satisfaction with work are both critical ingredients of continuous quality improvement and customer satisfaction [58,59]. Therefore, from the perspective of human motivation, the employees’ fulfillment of needs such as belonging, identity, and recognition from leaders and team members, can thus enhance their job satisfaction and promote their quality of working life [13]. Especially in the hospitality sector, these satisfied employees tend to have more willingness to stay in the organization, to participate in the TQM activities, to find solutions for quality improvement, and to contribute their efforts to assist their leaders and coworkers in meeting the organizational quality goal, thus helping the company to satisfy customers and create profitability [36,37,60]. For instance, Collins [33] undertook an empirical study in the service industry, and the results demonstrated the importance of high-quality LMX, with regard to employee turnover intention and job satisfaction. Erdogan and Enders [61] reported that positive relationships among LMX, job satisfaction, and job performance, and a high level of supervisors’ perceived organizational support, could enhance these relationships. Liden et al. [6] found that TMX is directly related to organizational commitment, job performance, and job satisfaction, in a field investigation of 337 employees. In addition, Cowling and Newman [46] revealed that job satisfaction is positively related to employee TQM involvement, based on a survey of service organizations in the UK. Accordingly, we posit that both high-quality LMX and high-quality TMX can contribute to the development of employee TQM involvement, through job satisfaction.

**Hypothesis** **11.** *LMX has a positive indirect effect on employee TQM involvement, via job satisfaction.*

**Hypothesis** **12.** *TMX has a positive indirect effect on employee TQM involvement, via job satisfaction.*

## 3. Methodology

### 3.1. Participants and Procedures

The participants in this study were collected from frontline employees in international tourist hotels in Taiwan. These frontline employees include members from the Front Office, Food and Beverage, and Rooms departments, who have the most direct contact with customers and have more responsibility to make decisions, depending on their own work situation. Thus, their involvement in TQM can help to increase customer satisfaction, raise customer switching costs and decrease advertising costs, and lead to business performance. Moreover, these hotels have successfully established the atmosphere to support employees in having good relationships with leaders and team members, and employees are also encouraged to participate in improving working processes and service quality. In addition, the data were collected during the respondents’ work hours, and they were ensured that their responses would remain confidential. We followed Brislin’s translation–back-translation procedure [62], and the items of our survey were first translated from English to Chinese and then translated back to English by separate management and bilingual scholars, to ensure correct meanings and equivalent translations. As can be seen in Supplementary Materials, responses were made using a seven-point Likert-type scale (e.g., 1, “strongly disagree”, to 7, “strongly agree”), with higher scores indicating more support for the item. Additionally, the common-method variance (CMV) might represent the variance in data attributed to the measurement method utilized, rather than the actual constructs represented by the measurements [63]. This variance leads to an erroneous internal consistency, giving rise to an appearance of correlation among variables, which is, in fact, generated by their mutual methodological source. To lower the concern with CMV, we asked employees to answer a number of items that contained questionnaires regarding their LMX, TMX, self-efficacy and job satisfaction, while direct leaders or supervisors assessed their TQM-involvement behavior.

Quota sampling was utilized as a non-probabilistic technique, to gather a representative dataset. The distribution of international tourist hotels in Taiwan across various regions, in 2020, was as follows: northern (53 percent), central (7 percent), southern (26 percent), eastern (9 percent), and other areas (5 percent). Correspondingly, the allocation of questionnaires mirrored this geographic distribution, with 15 hotels in the northern region, 2 in the central region, 7 in the southern region, 3 in the eastern region, and 1 in other areas. Working in collaboration with the Human Resources department, the survey questionnaires were distributed to all full-time front-line employees and to their direct leaders or supervisors in May 2020. To ensure clarity of research objectives, protect participant privacy, and establish contact points within each hotel, a cover letter accompanied the survey. Each employee received a stamped, self-addressed envelope, to facilitate the return of the completed questionnaires. These full-time employees are deeply engaged in their roles, often collaborating extensively with other full-time team members. Their extended tenure with the company often results in a strong sense of attachment to the company’s brand, making them a representative sample of the broader front-line employee population. Overall, out of the 1000 questionnaires, data in Table 1 were collected from 811 employees in 28 international tourist hotels, with a response rate of 81.1%. On average, there were 29 valid questionnaire responses received from front-line employees, in each hotel. The respondents in the main study were 54% female and 46% male, with their ages ranging from 21 to 63, with the average tenure in the organization being 5.2 years. In addition, 15% of the respondents had master’s degrees, 70% had bachelor’s degrees, and 15% had a high-school education.

### 3.2. Measures

LMX. LMX was assessed using the seven-item scale from Graen et al. [11]. Sample items are “My leader would be personally inclined to help me solve problems in my work”, and “I have enough confidence in my leader that I would defend and justify his or her decisions if he or she were not present to do so”. Cronbach’s alpha was 0.91 (alpha > 0.70), suggesting good reliability of the overall measurement.

TMX. TMX was measured using six items adapted from Seers et al. [5]. Three items asked about the individual’s contributions to the team, and the other three asked about what the individual received from the team. Sample items are “I will help finish work that had been assigned to others”, and “Other members of my team will help finish work that was assigned to me”. Cronbach’s alpha was equal to 0.85 (alpha > 0.70), revealing satisfactory reliability.

Self-efficacy. Four items from Bandura and Cervone [52] were used to determine employee self-efficacy. Sample items are “I could have handled a more challenging job than the one I will be doing”, and “My past experiences and accomplishments increase my confidence that I will be able to perform successfully in this organization”. Cronbach’s alpha for the measurement of self-efficacy was 0.80 (alpha > 0.70), indicating good reliability for this construct.

Job satisfaction. Job satisfaction was measured using three items based on the Michigan Organizational Assessment Questionnaire [64]. Sample items are “In general, I like my job”, and “All in all, I am satisfied with my job”. We calculated the stability for the three items, and Cronbach’s alpha was 0.85 (alpha > 0.70), showing satisfactory reliability.

TQM involvement. TQM involvement was assessed using the six-item scale from Fotopoulos et al. [47]. Sample items are “This employee participates in the decision-making process”, and “This employee takes part in designing quality improvement activities”. Cronbach’s alpha was 0.82 (alpha > 0.70), suggesting good reliability.

### 3.3. Analysis Strategy

We employed structural equation modeling (SEM), using AMOS 26.0, to evaluate the hypothesized model, utilizing maximum likelihood estimation [65]. Following Anderson and Gerbing’s [66] comprehensive two-step strategy for model examination, we initially subjected the measurement model to a confirmatory factor analysis (CFA). In addition, the internal-consistency reliability and convergent validity were also examined, using CFA. Finally, we investigated the causal relationships of LMX, TMX, self-efficacy, job satisfaction and TQM involvement in the structural model, using SEM analyses.

### 3.4. Confirmatory Factor Analyses

As discussed earlier, CFA was conducted to examine the proposed measurement model, and the fit indices were χ^2^ = 1479.68, df = 289, χ^2^/df = 5.12; GFI = 0.91; NFI = 0.93; IFI = 0.95; CFI = 0.95, RMSEA = 0.05, and SRMR = 0.05, which showed that the hypothesized five-factor model (LMX, TMX, self-efficacy, job satisfaction and TQM involvement) fits the main data well. We then explored the internal-consistency reliability, and convergent validity of the measurement model. As can be seen in Table 2, the results revealed that the composite reliability (CR) of each measure ranged from 0.84 to 0.92, better than the 0.60 CR standard value, and thus provided support for internal-consistency reliability of all measures [67,68]. Meanwhile, the items’ factor loadings in the five-factor measurement model were all significant (all *p* values < 0.001), which provided preliminary evidence for convergent validity [66]. Moreover, the average variance extracted (AVE) of each measure ranged from 0.53 to 0.67, over the 0.50 AVE standard value, and offered support for convergent validity, as well [67,68]. Furthermore, we also conducted Harman’s single-factor test, to better understand the influences of common-method variance (CMV) on the measurement model [63], and the results revealed that less than one-third (29.96%) only of the majority variance was explained by the first factor. As a result, we concluded that CMV did not have serious influences on the proposed five-factor model.

### 3.5. Test of Hypotheses

**Figure 2:**
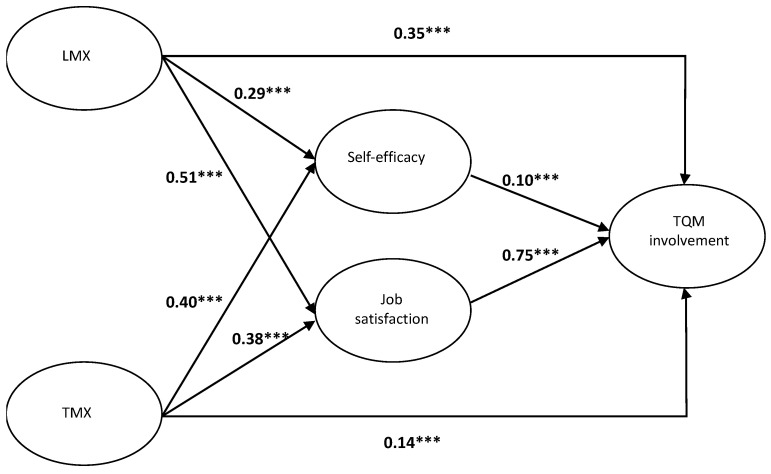
Structural equation modeling of the theoretical model. Note: *** means *p* < 0.001 (two-tailed).

This section shows the SEM analyses results for examining Hypotheses 1 through 6; and Baron and Kenny’s [69] suggestion of a causal steps strategy was followed, to test the condition of mediation. As for Hypothesis 1, Figure 2 reveals that the result of the direct effect of LMX on TQM involvement is positive and significant (standardized direct effect = 0.35, *p* < 0.001). Therefore, Hypothesis 1 is supported. As predicted in Hypothesis 2, the results in Figure 2 show that TMX has a significant positive relationship with TQM involvement (standardized direct effect = 0.14, *p* < 0.001). Thus, Hypothesis 2 is supported. As predicted in Hypothesis 3, the results in Figure 2 show that LMX has a significant positive relationship with self-efficacy (standardized direct effect = 0.29, *p* < 0.001). Accordingly, Hypothesis 3 is supported. As predicted in Hypothesis 4, the results in Figure 2 show that TMX has a significant positive relationship with self-efficacy (standardized direct effect = 0.40, *p* < 0.001). Therefore, Hypothesis 4 is supported. As predicted in Hypothesis 5, the results in Figure 2 show that LMX has a significant positive relationship with job satisfaction (standardized direct effect = 0.51, *p* < 0.001). Thus, Hypothesis 5 is supported. As predicted in Hypothesis 6, the results in Figure 2 show that TMX has a significant positive relationship with job satisfaction (standardized direct effect = 0.38, *p* < 0.001). Accordingly, Hypothesis 6 is supported. As predicted in Hypothesis 7, the results in Figure 2 show that self-efficacy has a significant positive relationship with TQM involvement (standardized direct effect = 0.10, *p* < 0.001). Hence, Hypothesis 7 is supported. In addition, as predicted in Hypothesis 8, the results in Figure 2 show that job satisfaction has a significant positive relationship with TQM involvement (standardized direct effect = 0.75, *p* < 0.001). Therefore, Hypothesis 8 is also supported, and the mediating roles of self-efficacy and job satisfaction on the relationships between LMX and TQM involvement, as well as TMX and TQM involvement, were all preliminarily supported.

To further test the indirect effects of LMX and TMX on employee TQM involvement, bias-corrected percentile bootstrapping was thus performed at a 99% confidence interval, with 6000 bootstrap samples [70]. Following Preacher and Hayes’ suggestions [71], the confidence intervals of the upper and lower bounds were calculated, to examine whether the mediating effects of employee self-efficacy and job satisfaction were significant. As seen in Table 3, the results of the bootstrap tests show evidence of a positive and significant indirect effect of self-efficacy on the relationship between LMX and TQM involvement (standardized indirect effect = 0.03, *p* < 0.01), a positive and significant indirect effect of self-efficacy on the relationship between TMX and TQM involvement (standardized indirect effect = 0.04, *p* < 0.01), a positive and significant indirect effect of job satisfaction on the relationship between LMX and TQM involvement (standardized indirect effect = 0.38, *p* < 0.001), and a positive and significant indirect effect of job satisfaction on the relationship between TMX and TQM involvement (standardized indirect effect = 0.29, *p* < 0.001). Therefore, partial mediating roles of employee self-efficacy and job satisfaction in Hypotheses 9, Hypotheses 10, Hypotheses 11, and Hypotheses 12 are all supported. Results of hypothesis testing were presented in Table 4.

Finally, for SEM model comparisons in Table 5, the first comparison for the hypothesized model (partially mediated model 1) and alternative model 1 (full mediation) showed that the change in chi-square/df value is significant (Δχ^2^ (Δdf) = 772.36 (2), *p* < 0.001). The result revealed that the hypothesized model provided a significantly better fit than the alternative model 1. Moreover, the second comparison for the hypothesized model (partially mediated model) and alternative model 2 (partially mediated model 2, linking self-efficacy to job satisfaction), showed that the change in chi-square/df value is significant (Δχ^2^ (Δdf) = 1370.52 (1), *p* < 0.001). It revealed that the hypothesized model had a significantly better fit than the alternative model 2. Similarly, the third comparison for the hypothesized model (partially mediated model) and alternative model 3 (partially mediated model 3, linking job satisfaction to self-efficacy), revealed that the change in chi-square/df value is significant (Δχ^2^ (Δdf) = 1702.87 (1), *p* < 0.001). It thus demonstrated that the hypothesized model had a significantly better fit than the alternative model 3. Therefore, it indicates that the hypothesized model fits the data better than any other alternative models.

## 4. Discussion

The study’s findings have several theoretical and practical implications for the hospitality industry. The confirmation of the mediating roles of self-efficacy and job satisfaction sheds light on the underlying mechanisms affecting employee TQM involvement. This underscores the significance of fostering high-quality relationships between leaders and team members in promoting self-efficacy and job satisfaction, and, ultimately, effective TQM participation among employees. From a practical standpoint, organizations in the hospitality sector should concentrate on cultivating positive social exchanges between leaders, coworkers, and employees to enhance job satisfaction and self-efficacy, thereby encouraging active involvement in TQM practices. These findings contribute to a better understanding of the intricate relationships which are crucial for the successful implementation of TQM in the hospitality industry. The implications of these results for theory and practice are discussed below.

### 4.1. Implications for Theory and Research

The results of our study contribute to the literature in several ways. First, LMX means the vertical-exchange relationships between employees and their leader, while TMX represents the horizontal-exchange relationships among team members [11,12], and these effects of exchange relationships with leaders and coworkers on individuals are unique and independent [10]. The current study is the first empirical work to consider the integrated influences of the quality of LMX and TMX on employee TQM-involvement behavior, using SEM analyses. The results of this study support the relationships proposed in the theoretical model, which suggest that employee involvement and their relationships with leaders and coworkers are vital for continuous quality-improvement activities [58,59]. Specifically, LMX and TMX both have positive significant effects with regard to predicting employee involvement in TQM. This can help us to conclude that the roles of mutual trust among employees, leaders and teammates can be regarded as core values of physiological and psychological needs in TQM practices [13].

Second, the findings of this work provide a new perspective on the partial mediating roles of employee self-efficacy in the relationships between LMX and TQM involvement, as well as TMX and TQM involvement. Confirmed by Bandura’s social cognitive theory [18], the results of this study reveal that self-efficacy, an individual’s belief in their own competence to carry out an assigned task well, can facilitate employee involvement in TQM activities. Moreover, employees with high self-efficacy can continually develop their abilities regarding quality-improvement activities, and thus enhance service performance [19,20]. Our finding thus concludes that employees could benefit from high-quality relationships with their leaders and coworkers, as this can raise their self-efficacy and participation regarding TQM activities. Therefore, the results suggest that LMX and TMX both help to create a social system that can positively influence employee TQM involvement, via the positive partial-mediating effects of self-efficacy.

Third, the results also demonstrate the positive effect that environmental context, as assessed by LMX and TMX, and individual context, as assessed through job satisfaction, may together have on employee TQM involvement in organizational settings. Interestingly, the support provided by leaders and coworkers is not only directly related to individual TQM involvement, but can also increase the overall satisfaction regarding their work, and so promote more involvement in TQM activities. The results of this work thus reveal that psychological states, such as job satisfaction and the relationship quality, both play vital roles in TQM activities in the work environment [13,72].

Overall, the results underscore the significance of self-efficacy, job satisfaction, and positive social exchanges (LMX and TMX) in fostering employee participation in Total Quality Management activities within the organizational environment. The findings highlight the fact that high-quality relationships with leaders and coworkers positively influence employee self-efficacy, and subsequently lead to increased involvement in TQM initiatives. Both LMX and TMX directly enhance TQM participation, and they also indirectly affect TQM involvement, by positively affecting self-efficacy and job satisfaction. This reveals a significant mediating effect. The study emphasizes the crucial role of self-efficacy, job satisfaction, and positive social exchanges in fostering employee engagement in TQM practices within an organizational framework.

### 4.2. Implications for Practice

The study’s outcomes hold significant implications for the hospitality industry. Recognizing the mediating roles of self-efficacy and job satisfaction highlights the critical mechanisms influencing employee involvement in Total Quality Management. This underscores the importance of fostering strong relationships between leaders, coworkers, and employees, to bolster self-efficacy, job satisfaction, and effective engagement in TQM practices. From a practical perspective, hospitality organizations should focus on nurturing positive social exchanges among their workforce, to cultivate higher job satisfaction and self-efficacy, thus encouraging active participation in TQM initiatives. These findings offer valuable insights into the complex relationships pivotal for successful TQM implementation in the hospitality sector.

Furthermore, the participation of employees in each quality process and improvement is the driver for the success of TQM, and can thus lead to business excellence [4]. In the hospitality industry especially, since frontline employees have the most direct contact with customers, their involvement in TQM can help the company to increase customer satisfaction and intention to repeat purchase. Accordingly, the findings of this study suggest that human resource departments in hotel companies should work to improve the relationships that their employees have with their supervisors and team members. Hoteliers and top managers can also attempt to foster a climate that can encourage supervisors to form useful exchanges with all their subordinates, and encourage staff to build high-quality relationships with both leaders and teammates. This concept of mutual trust is the core value of TQM, and helps to create a friendly environment for better relationship marketing and organizational learning [32]. Therefore, with the individuals’ physiological and psychological fulfillment of needs such as belonging, identity, and recognition from leaders and teammates, employees can achieve their satisfaction at work, as well as improving their quality of working life, and thus improve the quality of services for customers. In addition, hotel companies could provide effective quality training programs to promote employee self-efficacy in TQM practices and foster quality-improvement activities in organizations [73]. Accordingly, with employee TQM involvement in a synergistic way, hotels can thus enhance customer satisfaction, deliver high-value added services, and achieve business excellence [74].

In summary, the research highlights the significance of self-efficacy, job satisfaction, and favorable social connections (LMX and TMX) in stimulating employee participation in Total Quality Management endeavors within hospitality settings. The study accentuates the essential role of strong associations with both hospitality leaders and colleagues in positively shaping employee self-efficacy, and consequently enhancing their involvement in TQM initiatives.

### 4.3. Limitations and Future Research

This study has some limitations that are worth noting. One limitation is that it may have been affected by common-method variance (CMV), as the same employees self-reported their perceptions of LMX and TMX quality, as well as their levels of self-efficacy and job satisfaction [63]. Although we assessed employee TQM involvement from their direct leaders or supervisors, and the results of the Harman’s single-factor test also help to reduce this concern, with regard to the model presented in this work, future research should empirically and theoretically consider a broader or alternative social context of interpersonal exchange relationships, such as social network ties [75,76], and the implications of different types of exchange relationships with employee TQM involvement.

Another limitation of this study is the cross-sectional design, which neglected the causal relationships among the independent and dependent variables, since reverse causality between these could not be excluded. For instance, employees with different values might rank leaders differently. In TQM practices, high-self-efficacy employees might develop more high-quality LMX and TMX with their leaders and coworkers, while high-quality LMX and TMX relationships could enhance more individual self-efficacy. Longitudinal studies or alternative causal models should be considered in future research, to examine the directionality and dynamics of these factors [51].

Third, the function and meaning of social exchange relationships may be quite different in Chinese and Western societies. In Chinese society, employees tend to act according to their social roles, and work to maintain good relationships with their leaders and teammates. However, in Western society, employees tend to maintain their independent selves, and focus on connections between their values and those of others. Future researchers are thus encouraged to use alternative relationship constructs, such as Guanxi, in different cultural contexts and to examine their influences on employee TQM involvement, in order to explore any interesting differences that may exist [77].

Finally, the connection between self-efficacy and job satisfaction has significant importance in organizational behavior. Self-efficacy, a fundamental component within social cognitive theory, significantly influences employees’ motivation, perseverance, and task performance. Furthermore, job satisfaction is a pivotal aspect of organizational behavior, impacting employee turnover intentions and overall performance. The continuous improvement of individual capabilities within Deming’s TQM approach is bolstered by self-efficacy, continuously fostering employee involvement in activities aimed at enhancing quality. In the hospitality sector, contented employees are more likely to remain with the organization, actively participate in TQM initiatives, contribute to solving problems, and aid the organization in reaching quality objectives and achieving profitability. Although the SEM comparison results indicate that both linking self-efficacy to job satisfaction (alternative model 2) and linking job satisfaction to self-efficacy (alternative model 3) exhibit a poorer fit with the data, compared to the hypothesized model, future studies might consider gathering data from diverse organizations or sources to gain a more comprehensive understanding of the intricate relationship between self-efficacy and job satisfaction.

## 5. Conclusions

In summary, this research extends the literature on integrating social exchange theory and social cognitive theory, by stressing the influences of the LMX and TMX reciprocal relationships on employee TQM involvement. The results suggest that employees with better relationships with their leaders and coworkers can facilitate their TQM involvement behavior through self-efficacy and job satisfaction, and also provide a clear understanding of the causal chain mechanism operating in such social relationships.

## Figures and Tables

**Table 1:** Demographic characteristics.

Demographic Information	Category	Number	Percentage	Accumulation
Gender	Female	438	54%	54%
	Male	373	46%	100%
Age	18–25	195	24%	24%
	26–35	259	32%	56%
	36–45	146	18%	74%
	46–55	130	16%	90%
	56–65	81	10%	100%
Education	High school	121	15%	15%
	University	568	70%	85%
	Master’s	122	15%	100%
Tenure	Less than 1 year	187	23%	23%
	1 to 5 years	389	48%	71%
	Above 5 years	235	29%	100%
Location area	Northern	15	53%	53%
	Central	2	7%	60%
	Southern	7	26%	86%
	Eastern	3	9%	95%
	Others	1	5%	100%

**Table 2:** Confirmatory factor analysis of the measurement model.

Variable	Items	α	Variable	Factor Loadings	Means	S.E.	*t*-Value	AVE	CR
**LMX**	7	0.91	1. My leader would be personally inclined to help me solve problems in my work.	0.80	5.06	-	-	0.61	0.92
			2. My supervisor recognizes my potential.	0.71	4.98	0.05	21.01 (***)		
			3. My working relationship with my supervisor is effective.	0.86	4.86	0.04	28.17 (***)		
			4. My supervisor considers my suggestions for change.	0.76	5.09	0.04	23.96 (***)		
			5. My supervisor and I are suited to each other.	0.72	5.03	0.04	22.26 (***)		
			6. My supervisor understands my problems and needs.	0.81	4.92	0.04	26.02 (***)		
			7. I have enough confidence in my leader that I would defend and justify his or her decisions if he or she were not present to do so.	0.81	5.08	0.04	26.11 (***)		
**TMX**	6	0.85	1. I will help finish work that had been assigned to others.	0.72	4.91	-	-	0.56	0.88
			2. I will make suggestions about better work methods to other team members.	0.74	4.96	0.06	18.67 (***)		
			3. I will switch job responsibilities to make things easier for other team members.	0.71	5.04	0.07	16.99 (***)		
			4. Other members of my team will help finish work that was assigned to me.	0.75	5.01	0.06	18.79 (***)		
			5. Other members of my team will make suggestions about better work methods to me.	0.77	4.92	0.06	19.21 (***)		
			6. Other members of my team will switch job responsibilities to make things easier for me.	0.79	4.97	0.06	19.92 (***)		
**Self-efficacy**	4	0.80	1. I could have handled a more challenging job than the one I will be doing.	0.71	4.68	-	-	0.56	0.84
			2. I feel I am overqualified for the job.	0.80	4.72	0.09	17.06 (***)		
			3. I have confidence in my ability to solve problems.	0.76	4.60	0.09	16.54 (***)		
			4. My past experiences and accomplishments increase my confidence that I will be able to perform successfully in this organization.	0.73	4.58	0.09	16.24 (***)		
**Job**	3	0.85	1. In general, I like my job.	0.79	5.02	-	-	0.67	0.86
**satisfaction**			2. All in all, I am satisfied with my job.	0.86	5.09	0.04	26.21 (***)		
			3. In general. I don’t like working at this company (reverse-scored).	0.80	4.98	0.04	24.11 (***)		
**TQM**	6	0.82	1. This employee participates in the decision making process.	0.74	4.96	-	-	0.53	0.87
**involvement**			2. This employee participates in quality-improvement activities.	0.72	4.99	0.05	19.62 (***)		
			3. This employee takes part in designing quality-improvement activities.	0.70	4.84	0.06	18.54 (***)		
			4. This employee implements changes.	0.71	5.02	0.06	18.73 (***)		
			5. This employee takes initiatives.	0.79	4.82	0.04	21.37 (***)		
			6. This employee does not participate in quality-improvement activities (reverse-scored).	0.72	4.93	0.06	18.98 (***)		

Note: *** *p* < 0.001 (two-tailed); *n* = 811.

**Table 3:** Bootstrap analyses of the statistical significance of indirect effects.

Independent Variable	Mediator Variable	Dependent Variable	Standardized Indirect Effect	SE of Mean	99% CI Mean Indirect Effect (Lower and Upper)		Two-Tailed Significance
LMX →	SEE →	TQM	(0.29) × (0.10) = 0.03	0.09	0.01	0.04	**
TMX →	SEE →	TQM	(0.40) × (0.10) = 0.04	0.08	0.02	0.04	**
LMX →	JOB →	TQM	(0.51) × (0.75) = 0.38	0.09	0.06	0.09	***
TMX →	JOB →	TQM	(0.38) × (0.75) = 0.29	0.08	0.05	0.08	***

Note: (1) Standardized estimation of 6000 bootstrap samples; *n* = 811; ** *p* < 0.01; *** *p* < 0.001. (2) SEE = Self-efficacy; JOB = Job satisfaction; TQM = TQM involvement.

**Table 4:** Results of hypothesis testing.

Hypothesis	Path	Standardized Coefficient	Result
H1	LMX → TQM involvement	0.35 ***	Accepted
H2	TMX → TQM involvement	0.14 ***	Accepted
H3	LMX → Self-efficacy	0.29 ***	Accepted
H4	TMX → Self-efficacy	0.40 ***	Accepted
H5	LMX → Job satisfaction	0.51 ***	Accepted
H6	TMX → Job satisfaction	0.38 ***	Accepted
H7	Self-efficacy → TQM involvement	0.10 ***	Accepted
H8	Job satisfaction → TQM involvement	0.75 ***	Accepted
H9	LMX → Self-efficacy→ TQM involvement	0.03 **	Accepted
H10	TMX → Self-efficacy→ TQM involvement	0.04 **	Accepted
H11	LMX → Job satisfaction→ TQM involvement	0.38 ***	Accepted
H12	TMX → Job satisfaction→ TQM involvement	0.29 ***	Accepted

Note: ** *p* < 0.01; *** *p* < 0.001 (two-tailed); *n* = 811.

**Table 5:** SEM model comparison.

Model	χ^2^	df	χ^2^/df	Δχ^2^ (Δdf)	GFI	NFI	IFI	SRMR
***SEM models: all scales***								
Hypothesized model (partially mediated model 1)	1484.80	290	5.12	-	0.91	0.93	0.95	0.05
Alternative model 1 (full mediation)	2257.16	292	7.73	772.36(2) ***	0.88	0.88	0.89	0.06
Alternative model 2 (partially mediated model 2, linking self-efficacy to job satisfaction)	2855.32	289	9.88	1370.52 (1) ***	0.84	0.84	0.85	0.08
Alternative model 3 (partially mediated model 3, linking job satisfaction to self-efficacy)	3187.67	289	11.03	1702.87 (1) ***	0.83	0.83	0.84	0.08

Note: df = df for χ^2^, *p* = significance of χ^2^, *** *p* < 0.001 (two-tailed); *n* = 811.

## Data Availability

The data presented in this study are available on request from the corresponding author.

## Supplementary Materials

The following supporting information can be downloaded at: https://www.mdpi.com/article/10.3390/bs13121013/s1.
