# Supplementary material for: Do Social Exchange Relationships Influence Total-Quality-Management Involvement? Evidence from Frontline Employees of International Hotels
Source: Behav Sci (Basel). 2023 Dec 14;13(12):1013. doi: 10.3390/bs13121013 (PMC10740844; doi:10.3390/bs13121013)
Supplement: Supplementary file 1 [file behavsci-13-01013-s001.zip › behavsci-2669407-supplementary.pdf]

SECTION I

- **Please respond to the following questions**

LMX		Strongly Disagree		Neutral			Strongly Agree	
1.	My leader would be personally inclined to help me solve problems in my work.	1--	2--	3--	4--	5--	6--	7--
2.	I have enough confidence in my leader that I would defend and justify his or her decisions if he or she were not present to do so.	1--	2--	3--	4--	5--	6--	7--
3.	My working relationship with my supervisor is effective.	1--	2--	3--	4--	5--	6--	7--
4.	My supervisor considers my suggestions for change.	1--	2--	3--	4--	5--	6--	7--
5.	My supervisor and I are suited to each other.	1--	2--	3--	4--	5--	6--	7--
6.	My supervisor understands my problems and needs.	1--	2--	3--	4--	5--	6--	7--
7.	My supervisor recognizes my potential.	1--	2--	3--	4--	5--	6--	7--
TMX								
1.	I will help finish work that had been assigned to others.	1--	2--	3--	4--	5--	6--	7--
2.	I will make suggestions about better work methods to other team members.	1--	2--	3--	4--	5--	6--	7--
3.	I will switch job responsibilities to make things easier for other team members.	1--	2--	3--	4--	5--	6--	7--
4.	Other members of my team will help finish work that was assigned to me.	1--	2--	3--	4--	5--	6--	7--
5.	Other members of my team will make suggestions about better work methods to me.	1--	2--	3--	4--	5--	6--	7--
6.	Other members of my team will switch job responsibilities to make things easier for me.	1--	2--	3--	4--	5--	6--	7--
Self-efficacy								
1.	I could have handled a more challenging job than the one I will be doing.	1--	2--	3--	4--	5--	6--	7--
2.	My past experiences and accomplishments increase my confidence that I will be able to perform successfully in this organization.	1--	2--	3--	4--	5--	6--	7--
3.	I feel I am overqualified for the job.	1--	2--	3--	4--	5--	6--	7--
4.	I have confidence in my ability to solve problems.	1--	2--	3--	4--	5--	6--	7--

(continued)

Job satisfaction	Strongly Disagree			Neutral			Strongly Agree
1. In general, I like my job.	1--	2--	3--	4--	5--	6--	7--
2. All in all, I am satisfied with my job.	1--	2--	3--	4--	5--	6--	7--
3. In general. I don't like working at this company (reverse-scored).	1--	2--	3--	4--	5--	6--	7--
TQM involvement							
1. This employee participates in the decision making process.	1--	2--	3--	4--	5--	6--	7--
2. This employee participates in quality improvement activities.	1--	2--	3--	4--	5--	6--	7--
3. This employee takes part in designing quality improvement activities.	1--	2--	3--	4--	5--	6--	7--
4. This employee implements changes.	1--	2--	3--	4--	5--	6--	7--
5. This employee takes initiatives.	1--	2--	3--	4--	5--	6--	7--
6. This employee does not participate in quality improvement activities (reverse-scored).	1--	2--	3--	4--	5--	6--	7--

SECTION II General Information

1. Please select your gender. □ Female □ Male

2. Please indicate your age range.
   □ under 20 years □ 20 to 30 years □ 30 to 4 years
   □ 40 to 50 years □ 50 to 60 years □ 60 years or older

3. Please check your education level.
   □ High school □ Bachelor □ Master

4. Please select your tenure in this company.
   □ under 5 years □ 6 to 10 years □ 11 to 15 years
   □ 16 to 20 years □ 21 years or older

Any comments:

____

____

____

____
